# A retrospective review of oral cephalosporins versus fluoroquinolones for the treatment of pyelonephritis

**DOI:** 10.1371/journal.pone.0274194

**Published:** 2022-09-09

**Authors:** Kevin Lin, Yorgo Zahlanie, Jessica K. Ortwine, Wenjing Wei, Norman S. Mang, Bonnie C. Prokesch

**Affiliations:** 1 Department of Pharmacy, Parkland Health, Dallas, TX, United States of America; 2 Department of Pharmacy, Ochsner Medical Center, New Orleans, LA, United States of America; 3 Department of Pediatrics, University of Texas Southwestern Medical Center, Dallas, TX, United States of America; 4 Department of Internal Medicine, University of Texas Southwestern Medical Center, Dallas, TX, United States of America; 5 Director of Antimicrobial Stewardship, Parkland Health, Dallas, TX, United States of America; LSU Health Shreveport, UNITED STATES

## Abstract

**Background:**

The current Infectious Diseases Society of America guidelines for the treatment of acute uncomplicated pyelonephritis (AUP) advise caution when using oral beta-lactams due to concern for potentially inferior efficacy compared to fluoroquinolones (FQs) and trimethoprim-sulfamethoxazole; however, studies specifically evaluating the efficacy of oral cephalosporins (CPs) in AUP are limited.

**Objective:**

To assess the safety and efficacy of oral CPs versus FQs for the treatment of AUP.

**Design, setting and participants:**

This is a retrospective, chart review study conducted at a single-center, tertiary care hospital.

**Measurements:**

The primary endpoint was treatment failure within 30 days, defined as a change in antibiotic or return to ED or clinic due to persistent symptoms. Secondary endpoints included adverse drug reactions (ADRs) and *C*. *difficile* infection (CDI) within 30 days.

**Results:**

Of the 343 patients included in the study, treatment failure occurred in 54/338 (16.0%) patients and was similar between oral CPs and FQs (35/229 [15.3%] vs. 19/109 [17.4%]). A higher percentage of treatment failures were observed for third generation (3GC) and first generation (1GC) CPs compared to second generation CPs (2GC) (3GC: 15/65 [23.4%]; 1GC: 11/49 [22.4%]; 2GC: 9/115 [7.8%]). Documented ADRs were low (6/343 [1.7%]) and no cases of CDI were documented.

**Conclusions:**

Oral CPs appear to be as safe and effective as FQs for the treatment of AUP. Fewer treatment failures were noted with 2GCs as compared to 3GCs and 1GCs.

## Introduction

In 2016, the National Center for Healthcare Statistics identified urinary tract infections (UTIs) as one of the most common emergency department diagnoses among women of all ages with the estimated incidence of pyelonephritis in the United States ranging from 459,000 to 1,138,000 cases annually [1, 2]. Acute pyelonephritis refers to inflammation of the renal pelvis and kidney frequently caused by migration of bacteria up the urinary tract. *Escherichia coli (E*. *coli)* and other Enterobacterales represent the overwhelming majority of pathogens isolated in patients with pyelonephritis [3]. Clinical manifestations of the infection can range from localized symptoms to sepsis or septic shock. However, acute uncomplicated pyelonephritis (AUP), defined as pyelonephritis occurring in men or nonpregnant women without urological abnormalities or comorbidities, is typically not life-threatening if treated early in the disease course [4].

The most recent update of the international practice guidelines for the treatment of acute uncomplicated cystitis and pyelonephritis by the Infectious Diseases Society of America (IDSA) and European Society of Clinical Microbiology and Infectious Diseases (ESCMID) were published in 2011 and recommend fluoroquinolones (FQs) as first-line agents for pyelonephritis when prevalence of resistance is known to be ≤ 10% (level A-I recommendation) [3]. If local resistance rates are > 10%, a single initial intravenous (IV) dose of a long-acting antimicrobial such as ceftriaxone or a consolidated 24-hour dose of an aminoglycoside is recommended prior to starting the oral FQ course. Other therapeutic options include oral trimethoprim-sulfamethoxazole (TMP-SMX), also an A-I recommendation if known susceptibility, or alternatively an oral beta-lactam (β-lactam) [3]. Consistent with the guidelines, and due to the expanded spectrum of activity, high bioavailability and ease of dosing, FQs represent a significant proportion of outpatient antibiotic prescriptions with some estimates ranking them as the third most commonly prescribed antibiotic class [5]. However, the prevalence of FQ-resistant *E*. *coli* has increased to greater than 10%-30% nationally [6, 7]. At our institution, the most recent susceptibility data for *E*. *coli* isolated from the urine indicated 27% were resistant to ciprofloxacin. Though oral β-lactams are identified as alternative agents, the guidelines recommend caution when using them due to low quality evidence and studies showing decreased efficacy compared to FQs or TMP-SMX. Coupled with a lack of robust pharmacodynamic studies providing strong evidence of adequate drug concentrations in the renal parenchyma, the rationale for using oral CPs for the treatment of pyelonephritis is weak. However, when faced with limited oral treatment options, outpatient providers at our institution often elect to trial an oral β-lactam rather than admitting an otherwise clinically stable patient to the hospital for IV therapy. The purpose of this study was to assess the outcomes of these patients compared to those receiving first-line therapy and provide additional evidence for the safe and effective use of oral CPs for the treatment of AUP.

## Methods

A retrospective chart review was performed on all patients ≥ 18 years of age prescribed oral CPs or FQs for AUP upon discharge from the Emergency Department (ED) or observation unit at Parkland Hospital over a 12-month period from 9/1/2017 through 8/31/2018. Cases were defined based on attending provider chart documentation of “pyelonephritis” with study team providing further chart review to assess if the patient met criteria for AUP. For this study, AUP was defined as pyelonephritis needing observation for less than 48 hours or not requiring hospital admission. Additional criteria for inclusion were positive urine cultures with a speciated gram-negative pathogen which was susceptible to the antibiotic prescribed upon discharge from the ED or observation unit. Exclusion criteria included pregnancy, concurrent bacteremia, or presence of a urologic abnormality such as neurogenic bladder, ileostomy, or ileal conduit. If a patient had more than one pyelonephritis episode in the study period, only the first episode was included. The primary endpoint was treatment failure within 30 days from initial presentation to the ED, defined as the composite outcome of one or more of the following: return to an ED or clinic due to persistent symptoms, change in antibiotics due to persistent symptoms, or recurrence of UTI with the same organism. The 30 day time frame was used to differentiate treatment failure of original infection as opposed to onset of a new urinary infection. Persistent symptoms included dysuria, flank pain, fever, urgency, frequency, costovertebral angle (CVA) tenderness, and/or hematuria as documented in the chart by a medical provider. ED and outpatient clinic visits from several major hospitals in the area were also able to be reviewed through our electronic health record (EHR) to help assess for treatment failure. Secondary endpoints included documented adverse drug reactions (ADRs) and *Clostridioides difficile* infection (CDI) within 30 days of initial ED presentation.

Data collected included age, sex, β-lactam or FQ allergies, select non-urologic comorbidities (diabetes mellitus, present or previous cancer, gastrointestinal disease, liver disease, and immunocompromised status), urologic abnormalities (anatomic or functional urinary abnormality, chronic kidney disease, current urinary stones, continuous or intermittent foreign material in the urinary tract such as percutaneous nephrostomy tubes, ureteral stents, suprapubic catheters, or Foley catheters), urine culture results and antimicrobial susceptibilities, IV antibiotics administered during the ED visit, oral antibiotics prescribed at discharge, duration of therapy (DOT) categorized by ≤ 7 days or >7 days, treatment failure, adverse drug reactions, and CDI within 30 days of hospital presentation. Antibiotic susceptibilities were determined using the MicroScan WalkAway system with susceptibilities to all oral cephalosporins inferred from cefazolin susceptibilities per the Clinical and Laboratory Standards Institute (CLSI) M-100 guidance [8]. Immunocompromised status was defined as having primary immunodeficiency, autoimmune disease, or human immunodeficiency virus and/or receiving chemotherapy, biological agents, immunosuppressants, or steroids for >2 weeks prior to presentation. This study was approved by the UT Southwestern Institutional Review Board. As this was a retrospective study of medical records, all data were fully anonymized and the IRB waived the requirement for informed consent.

## Results

A total of 439 patients were screened and 343 were ultimately included in the study. Among these 343 patients, 229 were in the CP group and 109 in the FQ group. Patient characteristics are shown in Table 1. Age and race distributions were numerically similar between both groups. However, there was a higher rate of β-lactam allergies in the FQ group compared to that in the CP group (7.3% vs. 2.2%). There were no major differences between the various non-urologic comorbidities and urologic abnormalities in both cohorts. As expected, *E*. *coli* was the most common organism causing pyelonephritis in both groups (91.3% in CP group and 83.5% in FQ group). Despite there being 109 patients in the FQ group, the number of ESBL-producing and CRE organisms was unexpectedly small in this cohort (2 and 0 isolates, respectively). Initial IV antibiotic use, duration of IV antibiotics, oral antibiotic regimens, and total duration of antibiotic use is shown in Table 2. The majority of patients in both the CP and FQ groups received initial IV antibiotics (92.5% vs. 78%, respectively) and ceftriaxone was the predominant IV antibiotic administered. In the CP group, the most common oral antibiotic was cefuroxime (50.2%) followed by cefpodoxime (28.3%) and cephalexin (21.4%). Ciprofloxacin was the predominant agent in the FQ group (95.4%). The median total DOT in the CP group was 11 days (IQR 9–12.5 days) whereas in the FQ group it was 9 days (IQR 8–11 days). A significantly larger proportion of patients in the FQ group received ≤ 7 days of total antibiotic therapy (21.1% vs. 5.2%).

**Table 1:** Baseline characteristics.

	CP Group	FQ Group
	n = 229	n = 109
**Age–mean (±SD)**	42.41 (±14.8)	42.02 (±15.0)
**Female sex–n (%)**	205 (89.5)	88 (80.7)
**Hispanic–n (%)**	169 (73.8)	81 (74.3)
**Antibiotic allergy–n (%)**	9 (3.9)	13 (11.9)
β-lactam	5 (2.2)	8 (7.3)
FQ	1 (0.4)	0
**Non-urologic comorbidities–n (%)**		
Diabetes mellitus	77 (33.6)	25 (22.9)
Current or previous cancer	16 (7.0)	8 (7.3)
GI disease	8 (3.5)	5 (4.6)
Liver disease	8 (3.5)	8 (7.3)
Immunocompromising condition	9 (3.9)	10 (9.2)
**Urologic abnormalities–n (%)**		
Anatomic or functional urinary abnormality	8 (3.5)	9 (8.3)
CKD	11 (4.8)	3 (2.8)
Current urinary stones	24 (10.5)	10 (9.2)
Urinary tract foreign material	8 (3.5)	9 (8.3)
**Organism species–n (%)**		
E. coli	209 (91.3)	91 (83.5)
*Klebsiella* species	16 (7.0)	7 (6.4)
Others*	4 (1.7)	11 (10.1)
**ESBL organisms–n (%)**	0	2 (1.8)
**CRE organisms–n (%)**	0	0

* Others include *Enterobacter cloacae* (n = 3), *Proteus mirabilis* (n = 8), *Providencia rettgeri* (n = 2), *Pseudomonas aeruginosa* (n = 2)

Abbreviations–CP: Cephalosporin; FQ: Fluoroquinolone; GI: Gastrointestinal; CKD: Chronic kidney diseases; *E*. *coli*: *Escherichia coli*; ESBL: Extended-spectrum β-lactamase; CRE: Carbapenem-resistant *Enterobacteriaceae*.

**Table 2:** Antibiotic dose and duration of therapy.

	CP Group	FQ Group
	N = 229	N = 109
**Initial IV antibiotics–n (%)**		
Yes	212 (92.5)	85 (78.0)
**Initial IV antibiotic choice–n(%)**		
Ceftriaxone	210 (91.7)	82 (75.2)
Piperacillin/tazobactam	2 (0.8)	0
Ciprofloxacin	0	3 (2.8)
**Duration of IV antibiotics, days–n (%)**		
1	122 (53.3)	62 (56.9)
2	77 (39.3)	23 (21.1)
**Oral antibiotics and dosing–n (%)**		
Cephalexin	49 (21.4)	
500mg QID	16 (7.0)	
500mg TID	25 (10.9)	
500mg BID	6 (2.6)	
250mg QID	2 (0.9)	
Cefuroxime	115 (50.2)	
500mg BID	106 (46.3)	
250mg BID	9 (3.9)	
Cefpodoxime	64 (27.9)	
400mg BID	27 (11.8)	
200mg BID	30 (13.1)	
100mg BID	7 (3.1)	
Cefdinir 300mg BID	1 (0.4)	
Ciprofloxacin		104 (95.4)
500mg BID		104 (95.4)
Levofloxacin		5 (4.6)
750mg QD		4 (3.7)
500mg QD		1 (0.9)
**Duration of total antibiotics, days–n (%)**		
≤7	12 (5.2)	23 (21.1)
>7	217 (94.8)	86 (78.9)

Abbreviations–CP: Cephalosporin, FQ: Fluoroquinolone, QID: Four times daily, TID: Three times daily, BID: Two times daily, QD: Once daily

There was no difference in the rates of treatment failure between cohorts (CP vs. FQ: 35/229 [15.3%] vs. 19/109 [17.4%]). When stratified by reason for treatment failure, there was no difference between groups in the rate of ED or clinic return, change in antibiotic, or recurrent infection within 30 days (Table 3) and the primary reason for treatment failure in both groups was ED or clinic return (CP vs. FQ: 26/35 [74.3%] vs. 15/19 [78.9%]). Rates of treatment failure were stratified by antibiotics used in both the CP and FQ groups (Table 4). Cefpodoxime was associated with the highest rate of treatment failure (23.4%) in the CP group, followed closely by cephalexin (22.4%), while the lowest rate was observed with cefuroxime (7.8%). In the FQ group, three out of the five patients who were treated with levofloxacin had treatment failure (60%) compared to only 16/104 (15.4%) of the patients who received ciprofloxacin. Safety endpoints of ADRs and CDI were low and not different between groups (Table 3].

**Table 3:** Primary outcome and safety endpoints.

	CP Group	FQ Group
	N = 229	N = 109
30-Day treatment failure—n (%)	35 (15.3)	19 (17.4)
ED or clinic return	26 (11.4)	15 (13.8)
Change in antibiotic	6 (2.6)	6 (5.5)
Recurrent infection	9 (3.9)	4 (3.7)
ADR—n (%)	3 (1.3)	3 (2.8)
CDI—n (%)	0	0

Abbreviations–CP: Cephalosporin, FQ: Fluoroquinolone, ADR: Adverse drug reaction, CDI: *Clostridioides difficile* infection.

**Table 4:** Treatment failure by antibiotic.

**CP Group–n/N (%)** *			
Cephalexin	Cefuroxime	Cefpodoxime	Cefdinir
11/49 (22.4)	9/115 (7.8)	15/64 (23.4)	0/1 (0)
**FQ Group–n/N (%)**			
Ciprofloxacin		Levofloxacin	
16/104 (15.4)		3/5 (60)	

* Percentages of treatment failure are relative to the proportion of each antibiotic in the corresponding group

Abbreviations–CP: Cephalosporin; FQ: Fluoroquinolone.

## Discussion

The use of intravenous cephalosporins for community-onset acute pyelonephritis as initial treatment followed by an appropriately targeted oral antibiotic such as a FQ or TMP-SMX for a total duration of 7–14 days has demonstrated high clinical cure rates of ≥95% in several studies [9–11]. As rates of resistance to both FQs and TMP-SMX continue to increase, oral β-lactams are often the only remaining therapeutic option for pyelonephritis which might avoid hospital admission and allow for outpatient treatment of an otherwise clinically stable patient.

One of the primary concerns with using oral β-lactam agents is that despite being primarily eliminated via the kidneys, many of the agents in this class have limited bioavailability, potentially impeding the achievement of adequate renal tissue concentrations. For example, cefuroxime, cefpodoxime, and cefdinir have a bioavailability of 37%-52%, 50%, and 16%-21%, respectively [12–14]. In addition, few studies have explored the feasibility of oral cephalosporins for the treatment of Enterobacterales from a pharmacokinetic/pharmacodynamic (PK/PD) perspective. The pharmacodynamic target for efficacy of cephalosporins is the fraction of time above the organisms minimum inhibitor concentration (*ƒ*T>MIC) with the goal %*ƒ*T>MIC of 40–50%. Cattrall et al. sought to determine if there was a PK/PD basis for using oral antibiotics in the treatment of pyelonephritis, however the only cephalosporin evaluated was cephalexin [15]. Using a PK model to assess efficacy and minimum effective dose, the investigators found that achieving a 90% cumulative fraction of response (CFR) based on the European Committee on Antimicrobial Susceptibility Testing (EUCAST) breakpoint required cephalexin to be dosed at 3500mg every 6 hours which far exceeds the recommended daily maximum of 4000mg per day [15]. In a similar study by Rodriguez-Gascon et al., PK/PD analysis of cefuroxime and cefixime were conducted using Monte Carlo simulations to estimate CFR based on standard dosing regimens for these antibiotics. Results of their modeling showed that using a 90% probability of target attainment with %*ƒ*T>MIC 40% was achieved only when cefuroxime was dosed at 500mg every 8 hours for Enterobacterales with a MIC ≤1 mg/L (CLSI breakpoint ≤4 mg/L, EUCAST breakpoint ≤16 mg/L) and cefixime 400mg every 12 hours for a MIC ≤1 mg/L [16]. While the CLSI does not make specific comments on the utility of oral cephalosporins, it outlines that the urinary cefazolin breakpoint for Enterobacterales should be used as a surrogate to predict activity for most oral cephalosporins, including cephalexin, cefuroxime, and cefdinir. Moreover, while specific breakpoints exist for some oral cephalosporins and Enterobacterales, these may not be routinely available on standard susceptibility testing platforms.

The 2011 IDSA guidelines stated that evidence supporting the use of oral β-lactams following IV therapy for the treatment of pyelonephritis is limited [3]. A randomized, controlled trial comparing oral ampicillin to TMP-SMX for the treatment of acute pyelonephritis which found higher rates of recurrence in the ampicillin arm, thus concluding that TMP-SMX was preferred to oral β-lactams [17]. Another randomized trial by Cronberg et al. compared oral ceftibuten to norfloxacin for pyelonephritis and found significantly higher clinical failure rates and less complete cure with ceftibuten [18]. Since the release of the IDSA guidelines in 2011, no additional randomized trials have directly compared oral β-lactams to oral FQs. However, several randomized trials have demonstrated high rates of favorable clinical outcomes with oral CPs following initial IV CPs which can be compared to historically reported outcomes using oral FQs. Sanchez et al. evaluated the effectiveness of a 10-day course of oral cefixime 400 mg daily following a single dose of ceftriaxone for acute pyelonephritis and found no difference in clinical cure when compared to IV ceftriaxone alone (92% vs. 91%) [19]. Similarly, a 2012 prospective, double-blind, randomized trial compared the clinical efficacy of IV ceftriaxone for an average of 3 days followed by oral cefditoren pivoxil to IV ceftriaxone alone. After a total of 10 days of therapy in each group, no significant difference in clinical cure was observed between patients in the IV and oral therapy group compared to only IV therapy group (100% vs. 95.1%, 95% CI –0.12 to 0.02) [20]. Finally, Sutton et al. conducted a large, retrospective cohort study demonstrating the success of oral β-lactams for the treatment of Enterobacterales bacteremia secondary to a urinary source [21]. The investigators compared 955 patients who received an oral β-lactam to 3134 patients who received a FQ or TMP-SMX and found similar 30-day all-cause mortality or 30-day recurrent bacteremia between each group (4.4% vs. 3.0%, adjusted RR 1.31 [95%CI, 0.87–1.95]]. Both groups received IV antibiotics for a median duration of 3 days prior to oral step-down and were treated for a median total duration of 14 days. Though these studies had several limitations, they provide additional evidence suggesting that short course IV therapy (1–3 days) followed by oral β-lactams are likely effective for the treatment of AUP, as outlined in our study [19–21].

The overall rate of treatment failure in our study (54/338 [16.0%]) is similar in comparison to previous trials, which reported failure rates between 0%-37% [14–17, 22]. A retrospective study by Vogler et al. evaluated oral CPs for pyelonephritis in discharged patients from a community hospital ED [23]. The primary endpoint was treatment failure within 30 days defined as either a repeat healthcare visit or antibiotic changes based on susceptibilities and was significantly higher in the FQ or TMP-SMX group compared to the CP group (23% vs. 0), mostly due to resistance [23]. More recently, Fosse et al. evaluated 30-day UTI recurrence rates in outpatients diagnosed with pyelonephritis and treated with either an oral CP, FQ or TMP-SMX [24]. The recurrence rates of 16% among patients receiving oral CP and 17% among patients receiving first-line therapy are nearly identical to those observed in our study. Our research serves as one of only a handful of studies directly comparing oral β-lactams to FQs for treatment of AUP and adds to the growing evidence that oral CPs may be an equally efficacious alternative to FQs.

Reported rates of ADRs in previous research on this topic ranged from 0% to 47% depending on the study definition and trial design [16, 18, 19, 22]. The observed frequency of ADRs in our study was 1.7% overall and no CDIs were documented. Given the retrospective nature of this study, assessment of safety events was difficult and relied on patient follow-up and provider documentation, possibly underestimating the true rates of ADRs. With increasing recognition of adverse effects associated with FQ use such as tendonitis, mental status changes, and CDI, the oral CPs are a potentially appealing alternative due to their lower risk of severe of ADRs.

The conclusion of this study is promising yet limited in several ways. The retrospective design introduces inherent bias into the selection of patients for inclusion. Due to the smaller sample size, differences between groups may not have been adequately captured and thus we did not specify confounders or perform subgroup analyses as we felt the study would be most helpful in a primarily observational capacity. The antibiotic dosing, frequency, and duration of therapy were not standardized and compliance to oral therapy could not be assessed. In addition, as the infections were uncomplicated, it is plausible that the initial doses of IV therapy may have been adequate in and of themselves to cure the infection and that any additional oral therapy did not greatly impact cure rates. Furthermore, the population included in the study may not be generalizable to more complicated cases which require hospitalization, have significant comorbid conditions or urologic abnormalities, or have a history of multidrug-resistant pathogens. Although the primary outcome pooled the results of patients receiving any oral CP, we did not find all oral CPs to be equivalent. Safety endpoints occurred infrequently in both groups and may be underestimated due to the retrospective nature. However, the design of our study helps eliminate certain potential confounding elements such as clinical failures due to drug resistance and can more directly address clinical efficacy. Furthermore, our ability to access medical records from other major hospitals in the city may help to capture a more accurate rate of ED returns, clinical failures, and safety events.

Based on our study’s findings, oral CPs appear to be as safe and effective as oral FQs for the treatment of acute uncomplicated pyelonephritis. There were significantly less treatment failures with 2GCs as compared to 3GCs and 1GCs. Although oral CPs are not strongly recommended by guidelines for the treatment of AUP, there appears to be a growing consensus among practitioners that oral CPs and other β-lactams are a reasonable choice for uncomplicated cases given their tolerability and increasing resistance to first-line agents. Additional studies would be helpful in clarifying optimal oral CP agent selection and dosing for AUP.

## References

[1] Health, United States, 2016. National Center for Health Statistics. May 2017. Available from: https://www.cdc.gov/nchs/data/hus/hus16.pdf#074. 28910066

[2] Johnson JR, Russo TA. Acute pyelonephritis in adults. N Engl J Med. 2018;378:48–59. doi: 10.1056/nejmcp1702758 29298155

[3] Gupta K, Hooten TM, Naber KG, et al. International clinical practice guidelines for the treatment of acute uncomplicated cystitis and pyelonephritis in women: a 2010 update by the Infectious Diseases Society of America and the European Society for Microbiology and Infectious Diseases. Clin Infect Dis. 2011;52(5):e103–e120. doi: 10.1093/cid/ciq257 21292654

[4] Bailey R. Uncomplicated acute pyelonephritis in young men. Nephrology. 1996;2: 281–283.

[5] Kabbani S, Hersh AL, Shapiro DJ, et al. Opportunities to improve fluoroquinolone prescribing in the United States for adult ambulatory care visits. Clin Infect Dis. 2018;67(1):134–6. doi: 10.1093/cid/ciy035 29373664 PMC6005119

[6] Talan DA, Takhar SS, Krishnadasan A, et al. Fluoroquinolone-resistant and extended-spectrum β-lactamase–producing *Escherichia coli* infections in patients with pyelonephritis, United States. Emerg Infect Dis. 2016;22(9):1594–603. 10.3201/eid2209.160148 PMC4994338 27532362

[7] Spellberg B, Doi Y. The rise of fluoroquinolone-resistant *Escherichia coli* in the community: scarier than we thought. J Infect Dis. 2015;212(12):1853–5. 25969562 10.1093/infdis/jiv279 PMC4655852

[8] CLSI supplement M100. 30^th^ edition. Wayne, PA: Clinical and Laboratory Standards Institute; 2020. Available at: http://em100.edaptivedocs.net. Accessed February 24, 2021.

[9] Chang U, Kim HW, Wie SH. Propensity-matched analysis to compare the therapeutic efficacies of cefuroxime versus cefotaxime as initial antimicrobial therapy for community-onset complicated nonobstructive acute pyelonephritis due to Enterobacteriaceae infection in women. Antimicrob Agents Chemother. 2015 May; 59(5): 2488–2495. doi: 10.1128/AAC.04421-14 25645837 PMC4394823

[10] Chang U, Kim HW, Wie SH. Comparison of second- and third-generation cephalosporin as initial therapy for women with community-onset uncomplicated acute pyelonephritis. Yonsei Med J. 2015; 56(5): 1266–73. doi: 10.3349/ymj.2015.56.5.1266 26256969 PMC4541656

[11] Chang U, Kim HW, Wie SH. Use of cefuroxime for women with community-onset acute pyelonephritis caused by cefuroxime-susceptible or -resistant Escherichia coli. Korean J Intern Med. 2016; 31(1): 145–55. doi: 10.3904/kjim.2016.31.1.145 26767868 PMC4712418

[12] Cefdinir. Lexi-Drugs. Lexicomp. Wolters Kluwer Health, Inc. Riverwoods, IL. Available at: http://online.lexi.com. Accessed August 3, 2018.

[13] Cefixime. Lexi-Drugs. Lexicomp. Wolters Kluwer Health, Inc. Riverwoods, IL. Available at: http://online.lexi.com. Accessed August 3, 2018.

[14] Cefpodoxime. Lexi-Drugs. Lexicomp. Wolters Kluwer Health, Inc. Riverwoods, IL. Available at: http://online.lexi.com. Accessed August 3, 2018.

[15] Cattrall JWS, Asin-Prieto E, Freeman J, et al. A pharmacokinetic-pharmacodynamic assessment of oral antibiotics for pyelonephritis. Eur J Clin Microbiol Infect Dis. 2019;38(12):2311–21. doi: 10.1007/s10096-019-03679-9 31494827 PMC6858297

[16] Rodriuez-Gascon A, Aguirre-Quinonero A, Canut-Blasco A. Are oral cefuroxime axetil, cefixime, and cefditoren pivoxil adequate to treat uncomplicated acute pyelonephritis after switching from intravenous therapy? A pharmacokinetic/pharmacodynamic perspective. Enferm Infecc Microbiol Clin (Engl Ed). 2020;38(7):306–11. 32085928 10.1016/j.eimc.2019.12.017

[17] Stamm WE, McKevitt M, Counts GW. Acute renal infection in women: treatment with trimethoprim-sulfamethoxazole or ampicillin for two or six weeks. A randomized trial. Ann Intern Med. 1987 Mar;106(3):341–5. doi: 10.7326/0003-4819-106-3-341 3492950

[18] Cronberg S, Banke S, Bergman B, et al. Fewer bacterial relapses after oral treatment with norfloxacin than with ceftibuten in acute pyelonephritis initially treated with intravenous cefuroxime. Scand J Infect Dis. 2001;33:339–43. doi: 10.1080/003655401750173922 11440218

[19] Sanchez M, Collvinent B, Miro O, et al. Short-term effectiveness of ceftriaxone single dose in the initial treatment of acute uncomplicated pyelonephritis in women. A randomized controlled trial. Emerg Med J. 2002;19:19–22. 11777865 10.1136/emj.19.1.19 PMC1725780

[20] Monmaturapoj T, Montakantikul P, Mootsikapun P, et al. A prospective, randomized, double dummy, placebo-controlled trial of oral cefditoren pivoxil 400 mg once daily as switch therapy after intravenous ceftriaxone in the treatment of acute pyelonephritis. Intl J Infect Dis. 2012;16:e843–9. 10.1016/j.ijid.2012.07.009 22951426

[21] Sutton JD, Stevens VW, Chang NN et al. Oral β-lactam antibiotics vs fluoroquinolones or trimethoprim-sulfamethoxazole for definitive treatment of Enterobacterales bacteremia from a urine source. JAMA Netw Open. 2020;3(10):e2020166. doi: 10.1001/jamanetworkopen.2020.20166 33030555 PMC7545306

[22] Hooten TM, Scholes D, Gupta K et al. Amoxicillin-clavulanate vs ciprofloxacin for the treatment of uncomplicated cystitis in women. JAMA. 2005;293:949–55. 15728165 10.1001/jama.293.8.949

[23] Vogler S, Pavich E. Pyelonephritis treatment in the community emergency department: cephalosporins vs. first-line agents. Am J Emerg Med. 2018. S0735-6757(18)30652–1. doi: 10.1016/j.ajem.2018.08.016 30119986

[24] Fosse PE, Brinkman KM, Brink HM, et al. Comparing outcomes among outpatients treated for pyelonephritis with oral cephalosporins versus first-line agents. Int J Antimicrob Agents. 2022. [Epub ahead of print]. doi: 10.1016/j.ijantimicag.2022.106560 35259485

